# *GUCY2D*-Associated Retinopathy: A Comparative Study Between Humans and German Spitz Dogs

**DOI:** 10.3390/vetsci12090879

**Published:** 2025-09-11

**Authors:** Bianca L. V. Guareschi, Juliana M. F. Sallum, Mariana V. Salles, João G. O. de Moraes, Mariza Bortolini, Carolyn Cray, Bret A. Moore, Carolina C. da Rosa, Fabiano Montiani-Ferreira

**Affiliations:** 1 Department of Veterinary Medicine, Federal University of Paraná (UFPR), Curitiba 80050-350, Brazil; biaguareschi@gmail.com (B.L.V.G.); bortolinimariza@gmail.com (M.B.); carolinacdarosa@gmail.com (C.C.d.R.); 2 Federal University of São Paulo (UNIFESP), São Paulo 04023-000, Brazil; juliana@pobox.com (J.M.F.S.); marivallim@yahoo.com.br (M.V.S.); 3 Cajuru University Hospital, Curitiba 80050-350, Brazil; jgyimoraes@terra.com.br; 4 School of Medicine, University of Miami Miller, Miami, FL 33136, USA; ccray@med.miami.edu; 5 Department of Clinical Sciences, Cornell University, Ithaca, NY 14853, USA; bretskimoore@gmail.com

**Keywords:** Leber’s congenital amaurosis, animal model, gene therapy

## Abstract

The anatomical and physiological similarities between human and canine eyes suggest that dogs may serve as a valuable model for studying retinopathies and developing future gene therapies. This study aims to evaluate the similarities and differences between humans with *GUCY2D* gene variants causing Leber’s congenital amaurosis (LCA) and a group of German Spitz dogs with hereditary retinopathy due to variants in the same gene, to assess their potential as an animal model for gene therapy research. A review of medical records, genetic testing, and ophthalmological examinations was conducted, including data such as age, genotyping, fundus photography, visual acuity (VA), fundus autofluorescence, optical coherence tomography (OCT), and electroretinography (ERG). Both groups presented subtle fundus abnormalities and severely reduced or absent ERG responses. In humans, OCT scans revealed decreased retinal thickness and structural alterations in the outer retinal layers. Similarly, the affected dogs exhibited focal neurosensory retinal detachments. The German Spitz model with *GUCY2D* variants shows significant parallels in retinal structure and functional impairment and may represent a promising candidate for preclinical gene therapy studies for LCA.

## Simple Summary

This study aimed to evaluate similarities between humans and German Spitz dogs, both of whom have vision problems caused by changes in the same gene. In humans, this condition is called Leber’s congenital amaurosis (LCA), which leads to severe vision loss from a young age. For this study, medical records, genetic tests, and eye exams from both humans and dogs were reviewed. The results showed that both groups had poor or no responses in eye function tests and changes in the back of the eye. Humans had thinner retinas and changes in certain layers, while dogs exhibited small areas where the retina was separated. These findings suggest that German Spitz dogs have eye problems that are very similar to those in humans with LCA. We propose that dogs could be a useful model to test new gene therapies for LCA in humans.

## 1. Introduction

The *GUCY2D* gene is responsible for the production of the retina-specific enzyme guanylate cyclase 2 expressed in the outer segment of photoreceptor cells, which is responsible for photoreceptor recovery in phototransduction. The function of the enzyme is decreased with reduced levels of cytoplasmic calcium levels (Ca ^2+^) [1].

In humans, variants in the gene coding for the retina-specific enzyme guanylate cyclase 1 exhibit two common phenotypes: autosomal recessive inheritance (AR) causing type I Leber’s congenital amaurosis (LCA), and autosomal dominant inheritance (AD) causing cone-rod dystrophy (CRD). Other, more rare phenotypes have also been described in association with the *GUCY2D* variant, such as central areolar dystrophy and early-onset retinitis pigmentosa [2]. Among the most severe and earliest congenital dystrophies of childhood, LCA represents approximately 9% of all patients [3] and *GUCY2D* variants are causal in 10–20% of those cases [2]. CRD starts in the first decade of life and affects the macula, and the main symptom is visual impairment with central scotoma. To date, 13 genes have been identified as being responsible for non-syndromic CRDs. *GUCY2D* variants are present in approximately 25% of cases and are limited to exon 13, which encodes the dimerization domain of guanylate cyclase. It is anticipated that additional genes will be identified in the future [2,4,5].

A condition analogous to LCA in humans was recently identified as an important cause of vision loss in domestic canines carrying a *GUCY2D* variant (*c.1598_1599insT* spontaneous frameshift homozygosis variant in exon 7) and exhibiting a form of progressive retinal atrophy (PRA). This was described in our previous work^6^ on the identification and genetic characterization of the *GUCY2D* variant in German Spitz dogs affected by early-onset PRA. In that initial report, we established the pathogenicity of a frameshift variant and documented its associated clinical phenotype. In the present study, we advance this line of research by employing the canine model in a comparative framework with humans carrying autosomal recessive *GUCY2D*-associated retinal dystrophies. This progression from genetic characterization to cross-species phenotypic modeling strengthens the translational bridge between veterinary and human ophthalmic genetics [6]. Anatomical similarities with the human eye, especially size and a retinal area with a high density of cones destined for high visual acuity, can make dogs a superior animal model for vision studies compared to rodents [7,8].

The aim of this study is to compare a group of German Spitz dogs exhibiting early-onset hereditary retinopathy with a group of humans carrying variants in the same gene and presenting the LCA phenotype. Furthermore, we will explore various factors that may support future translational ophthalmological research, including advancements in gene therapy.

## 2. Materials and Methods

### 2.1. Ethical Approval

The Ethics Committee of Health Sciences at UFPR approved the retrospective descriptive study conducted on humans on 6 April 2022 (54931221.4.0000.0102). The study involving German Spitz dogs received approval from the Committee on the Ethical Use of Animals (CEUA) of UFPR (050/2021) on 15 October 2021.

### 2.2. Patients

This study involved a review of medical records and ophthalmologic examinations of 10 human patients affected by a variant in the *GUCY2D* gene, conducted at the Institute of Ocular Genetics, São Paulo, SP, Brazil. All human participants underwent genetic testing, and only those with the autosomal recessive phenotype were included. Informed consent was obtained from all patients. A second comparative group consisted of 16 German Spitz dogs from a breeder, examined at the Veterinary Hospital of the Federal University of Paraná, which were affected by a genetic variant in the *GUCY2D* gene and displayed phenotypic signs of early-onset PRA. All dogs in the study group were either confirmed to be homozygous for the c.1598_1599insT variant (p.Ser534GlufsTer20) or were affected littermates with a clinically confirmed disease phenotype. The 16 dogs in the present study are the same individuals previously reported [6]. However, all of the clinical data presented here are entirely original.

### 2.3. Clinical Examination and Tests

In reviewing the medical records of human patients, the following information was considered: sex, age, genetic test (alleles and variant when available), clinical history, best corrected visual acuity improvement (BCVA) using the Snellen chart, and ophthalmologic examination. BCVA was considered extremely compromised when both light perception (LP) and hand movements (HM) were absent. Fundus photographs were analyzed and categorized by the same ophthalmologist with an emphasis on the presence of arteriolar attenuation, venous attenuation, optic disc pallor, and retinal macular changes. Some patients had also undergone additional tests such as fundus autofluorescence (FAF), electroretinography (ERG), optical coherence tomography (OCT), and visual field (VF) testing.

The diagnosis of PRA in the 16 German Spitz dogs was performed at the Veterinary Hospital of UFPR inclusive of a review of their medical records. The following data were evaluated: sex, age at diagnosis, type of genetic inheritance, visual acuity (assessed by menace response, dazzle reflex, pupillary light reflex, cotton ball test, and obstacle avoidance in both photopic and scotopic conditions), presence of nystagmus, and genetic test results. Each maze trial included three attempts, all conducted under standard room lighting. At the start of each attempt, the owner called the dog’s name and then remained silent to avoid unintentionally guiding the dog. If the dog did not complete the maze within one minute, the owner was instructed to call the dog again. Any dog that failed to complete the maze within 5 min was recorded as a failure.

Fundus photography was analyzed by the same ophthalmologist (BLGV, FMF) for the following parameters: presence of blood vessel attenuation, presence or absence of neurosensory retinal detachment (NRD), the area of the tapetum, presence or absence of tapetal hyperreflectivity, accumulation of pigment spots in the retina, and optic disc pallor. Dogs who were submitted to OCT had images evaluated by the same individual for total retinal thickness of the ventral retina in relation to the dorsal and thickness of the outer nuclear layer (ONL), and the presence or absence of NRD. ERGs were also performed and evaluated.

For OCT, pupil dilation was performed. Subsequently, 10 retinal cross-sectional images were obtained for each eye, covering tapetal, nontapetal, and peripapillary regions. Total retinal and outer nuclear layer (ONL) thicknesses were measured in the dorsal (2000–3000 μm from the dorsal edge of the papilla) and ventral (2000–3000 μm from the ventral edge of the papilla) retinal areas. In each region, measurements were taken from four adjacent cross-sections, avoiding retinal vessels [9], using the software provided in the Spectralis SD-OCT (Heidelberg Engineering©, Franklin, MA, USA).

Electroretinography (ERG) was performed at least 60 min following clinical examination and imaging. Pupils were dilated using 1% tropicamide eye drops (Mydriacyl, Alcon^TM^, Vernier, Switzerland) and 10% phenylephrine eye drops (Frumtost). In humans, ERGs were conducted following ISCEV standards. For dogs, two ERG protocols were used. The first protocol, performed on dark-adapted (20 min) conscious animals, involved a simple protocol using flash stimuli of 0.47 and 1 log cds/m^2^ (with each stimulus, an average of four flashes at 0.05 Hz was recorded). These “standard” and “high-intensity” stimuli both elicited a mixed rod–cone response. The second protocol used a preprogrammed ERG sequence. Ten ERGs in both protocols were recorded using a portable ERG unit equipped with a Mini Ganzfeld flash photostimulator with white LEDs (HMsERG VET System, OcuScience^®^, Henderson, NV, USA), positioned 1 cm from the corneal surface. A corneal contact recording electrode (ERG-Jet, Fabrinal SA, Neuchatel, Switzerland) served as the active electrode (placed following the application of 0.5% proparacaine hydrochloride ophthalmic solution USP; Alcon Laboratories for the first protocol). The reference and ground electrodes were platinum subdermal needles (Model E2, Grass Technologies, Borris, Ireland) placed 2 cm from the lateral canthus and over the dorsal cervical region, respectively. Electrode impedance was maintained at <5 kΩ, with a bandpass of 0.3–300 Hz [10,11].

## 3. Results

### 3.1. Humans

This group consisted of ten participants (five females, five males) affected by the disease-associated *GUCY2D* autosomal recessive gene variant. The ages ranged from 4 to 55 years (mean ± standard deviation = 18.2 ± 15.5 years). Human patients were identified as 01 to 10. Each individual is described with the respective genetic and ocular findings (Table 1). Patient 05 presented with keratoconus and two patients had strabismus (03 and 08).

**Figure 1:**
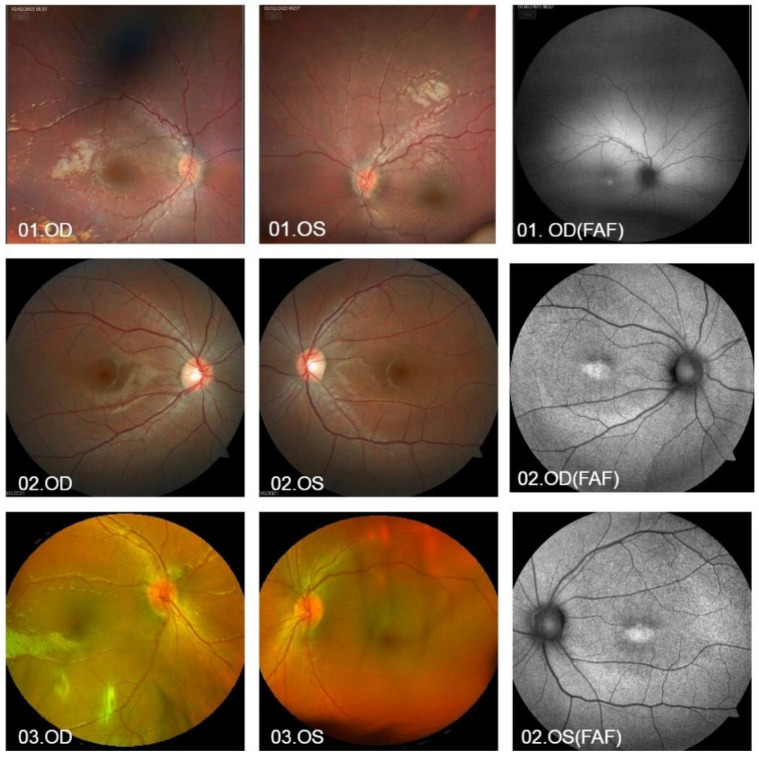
Ophthalmological findings of patients 01–03 in the human group. 01.OD and 01.OS: Fundus photographs show increased arteriolar tortuosity is present in OU. 01.OD(FAF): Auto fluorescence examination showing hyper-autofluorescence in OD. 02.OD and OS: Color fundus photography without notable abnormalities. 02.OD(FAF) and OS(FAF): Autofluorescence examination of the same patient showing hyper-autofluorescence in OU. 03.OD and OS: Color fundus photography without alterations. OU: both eyes, OD: right eye, OS: left eye.

Twenty percent of patients (2/10) presented with arteriolar attenuation (04 and 05) and two patients presented with increased arteriolar tortuosity (01 and 08, who were siblings) (Figure 1, Figures S1 and S2). Venous attenuation and optic disc pallor were not observed in any of the patients. Patients 04 and 10 exhibited a significant reduction in sensitivity macula and decreased sensitivity in all quadrants of VF (Figure S3). Extremely low BCVA was observed in seven patients (7/10) and nystagmus was described in five patients (5/10). A history of consanguinity was described in three patients (3/10).

**Figure 2:**
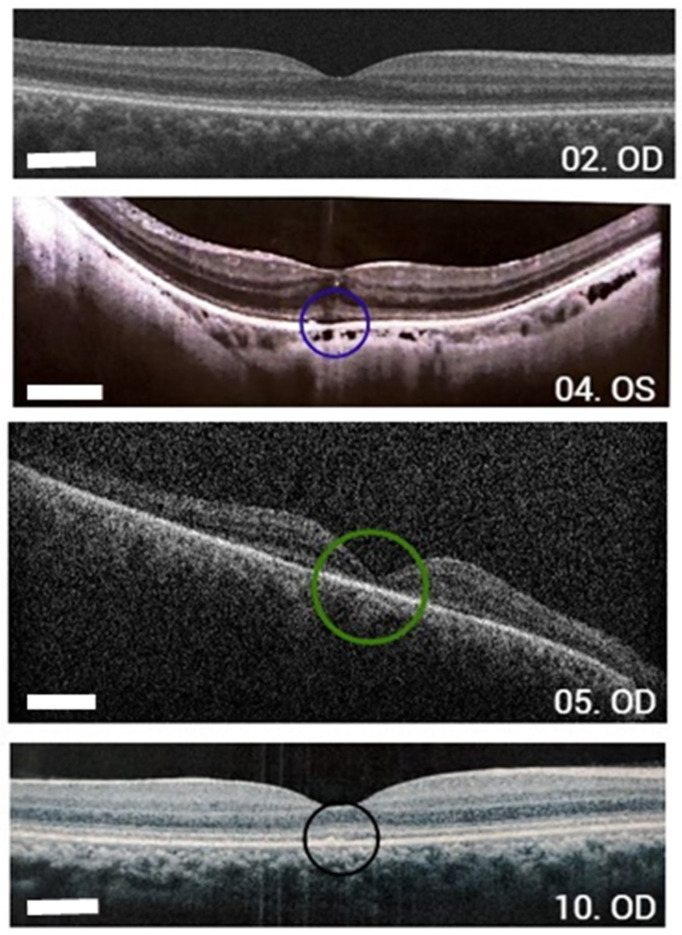
Macula OCT scans from human group. 02. OD: Macula OCT scan of patient 02 shows an unaltered inner, outer retinal architecture and choriocapillary. 04. OS: Macula OCT scan of patient 04 shows reduced reflectivity of the RPE and the ellipsoid zone with point interruption in the fovea (blue circle). 05. OD: Macula OCT scan of patient 05 indicates an absence of the ellipsoid zone foveal and perifoveal RPE (green circle). 10. OD: Macula OCT scan of patient 10 displays foveal outer retinal irregularities (black circle). OCT: optical coherence tomography, RPE: retinal pigment epithelium, OD: right eye, OS: left eye. Bar size: 250 µm.

In patients evaluated with an autofluorescence exam, all exhibited changes with the presence of hyper-autofluorescence (patients 01, 02, 04, 06, 08, and 10). Patient 02 presented with normal OCT of the macula and optic nerve. The OCT of the macula of patients 05 showed foveal outer retinal atrophy; patient 04 had foveal focal outer retinal defect and extrafoveal outer retinal rarefaction; and patient 10 had foveal outer retinal irregularity (Figure 2).

Patients 03, 09, and 10 presented an ERG exam with an absence of cone and rod responses. Patient 04 and 10 presented with visual field results (using Humphrey^®^ 24-2, Zeiss, Hebron, KY, USA) with a significant reduction in macula sensitivity and decreased sensitivity in all quadrants.

### 3.2. German Spitz Dogs

Group 2 consisted of 16 German Spitz dogs (6 females, 10 males) affected by early-onset retinopathy caused by the AR variant in the *GUCY2D* gene, with ages ranging from 1.5 to 22 months (mean ± standard deviation = 5.2 ± 5.4 months). The dogs were numbered 1–16. The age at diagnosis, fundus photography, ERG, and OCT, as well as the results of genetic testing, are reported in Table 2.

The genetic inheritance observed for all dogs was AR. The genetic testing of affected dogs revealed a *c.1598_1599insT* spontaneous frameshift homozygosis variant in exon 7 of the *GUCY2D* gene, which produces the p.Ser534GlufsTer20 protein. The remaining dogs had the same phenotypic condition and all were siblings.

All dogs failed the cotton ball test and obstacle avoidance in photopic and scotopic conditions in the exam room and thus were considered to have low visual acuity. Oscillatory nystagmus was present in 13 of 16 dogs (81.25%). In all dogs, there was no response in scotopic and photopic ERGs exam through one year of age.

**Figure 3:**
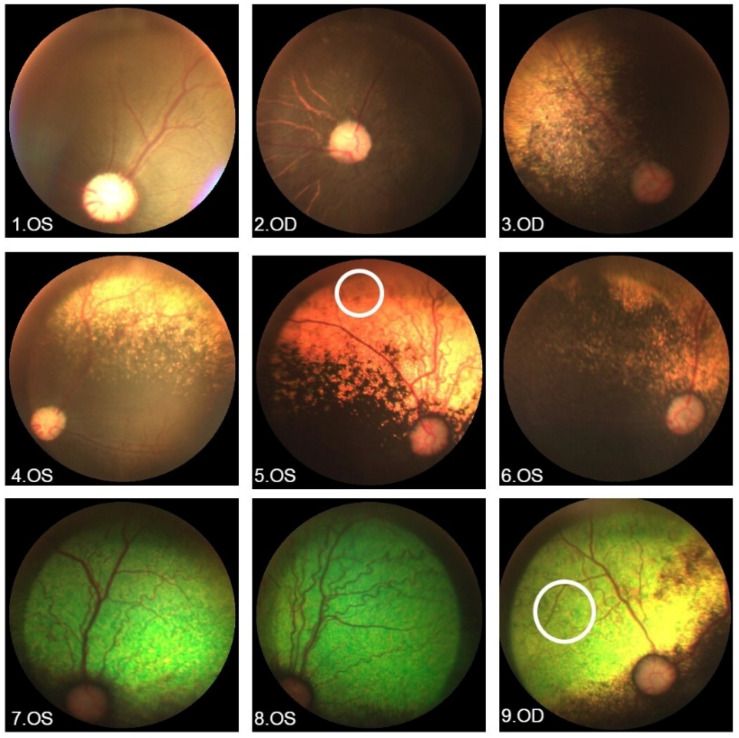
Ophthalmological findings in the dog study group. 1. OS: Arteriolar attenuation and absent tapetum. 2. OD: Arteriolar attenuation, hypoplastic tapetum, slight pigmentary mobilization, and choroidosis. 3. OD: Mild arteriolar attenuation and hypoplastic tapetum with hyperreflectivity. 4. OS: Mildly affected with hypoplastic tapetum with hyperreflectivity. 5. OS: Increased arteriolar tortuosity, hypoplastic tapetum with hyperreflectivity, and presence of neurosensory retinal detachment (circle). 6. OS: Arteriolar attenuation and hypoplastic tapetum with hyperreflectivity. 7. OS: Increased arteriolar tortuosity and tapetal hyperreflectivity. 8. OS: Increased arteriolar tortuosity and tapetal hyperreflectivity. 9. OD: Arteriolar attenuation, hypoplastic tapetum with hyperreflectivity, and presence of neurosensory retinal detachment (circle). OS: left eye, OD: right eye.

Fundus photography (Figure 3 and Figure S4) showed slight arteriolar attenuation in seven dogs and increased tortuosity in four dogs. Optic nerves with a normal pink appearance and retinal veins of normal caliber were present in all dogs. Three of the dogs had slightly mild pigmentary dispersion and some degree of choroidal abnormalities (2, 13, 14). The area of the tapetum was reduced or hypoplasic in seven dogs and completely absent in four dogs. Hyperreflectivity of the tapetum was observed in eight dogs.

**Figure 4:**
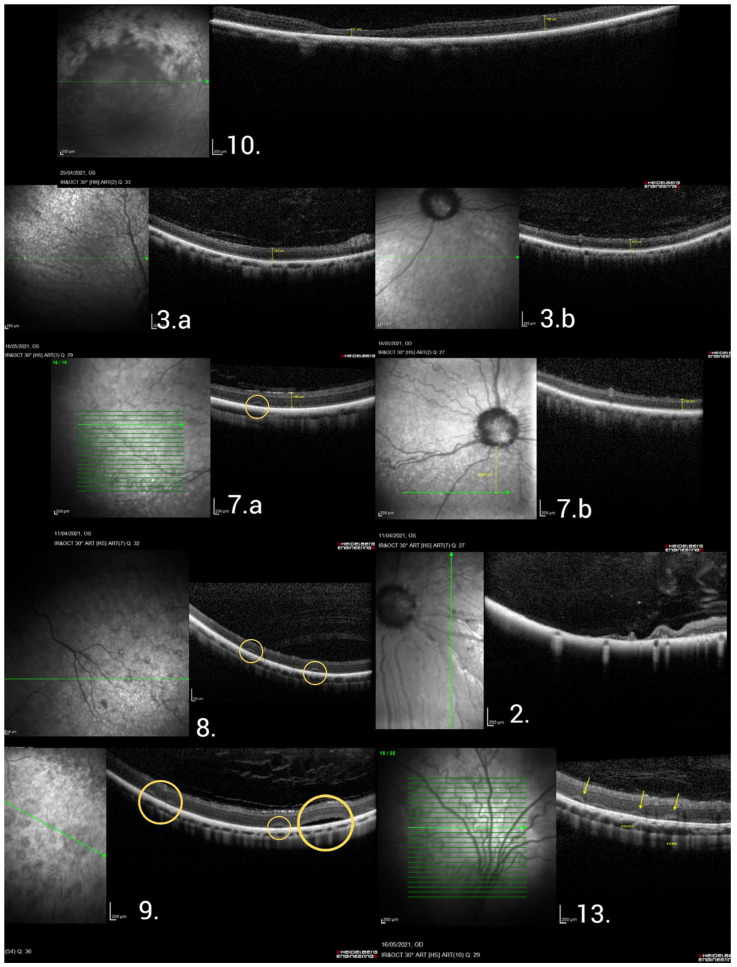
Macula OCT scans from the dog study group. 10: Local atrophy of the outer retina with collapse of the inner retina reducing the thickness to 87 μm. 3.a and 3.b: Ellipsoid zone rarefaction and measurements of dorsal retinal thickness (3.a) of 154 μm relative to a ventral retinal thickness (3.b) of 147 μm. 7.a and 7.b: Ellipsoid zone rarefaction, focal detachments of the neurosensory retina (NRD) (yellow circle), and measurements of dorsal retinal thickness (7.a) of 186 μm relative to a ventral retinal thickness (7.b) of 134 μm. 8: Presence of two focal NRDs (yellows circles). 2: Complete atrophy of all ventral retinal layers in relation to the dorsal. 9: Presence of four focal NRDs (yellow circles). 13: Ellipsoid zone rarefaction and five focal NRD (yellow arrows). OCT, optical coherence tomography; NRD, detachment of the neurosensory retina. Bar sizes on the left corner: 200 µm.

Seven dogs (2, 3, 7–10, and 13) had OCT exams, in which focal NRD (Figure 4) was observed in four (7–9, 13) and reduction of the ventral retina in relation to the dorsal retina in all cases. An additional three dogs had focal NRDs detected in fundus photography (1, 4, and 5).

The OCT scans (dogs and age at the time of the exam present in Figure 4) of dogs 3, 7, 10, and 14 showed an outer nuclear layer (ONL) medium thickness of 42 μm, 47.5 μm, 56 μm, and 64.5 μm, respectively, with ellipsoid zone absence in one of the dogs (10) and ellipsoid zone rarefaction in dogs 3, 7, and 13.

## 4. Discussion

The present study extends our earlier work describing the *GUCY2D* variant in German Spitz dogs by moving beyond variant discovery and clinical description to a direct comparative analysis with human LCA patients. This stepwise approach from defining the molecular defect in a naturally occurring animal model to integrating it into a comparative translational context provides a more comprehensive understanding of disease mechanisms [6].

To provide a clearer overview of the similarities and differences between the human and dog cohorts, we compiled a side-by-side comparison of their main clinical, electrophysiological, and fundus imaging characteristics (Table 3). This comparative format allows for a more immediate visualization of overlapping features, such as early onset, severe visual impairment, and absence of both cone and rod responses on ERG, as well as species-specific findings, including retinal pigment epithelium changes in humans and tapetal abnormalities in dogs. Table 3 serves as a framework for the subsequent discussion, in which these shared and distinct features are analyzed in greater detail to explore their implications for disease understanding and the translational applicability of the dog model.

A previously published study in humans reported that a total of 144 variants have been reported in the *GUCY2D* gene, of which 127 (88%) are associated with LCA and 13 (9%) cause CRD [12]. The variants found in the human group were AR with phenotypic expression of LCA, and our results correspond to those published in relation to the prevalence of the AR phenotype. All of the gene variants related to the *GUCY2D* gene in humans described in this retrospective study have been previously identified [13].

Consanguinity is present in more than 10% of humans with hereditary retinal dystrophy (RD), and, more specifically in LCA, it accounts for 9% of cases, and, in early RD, it accounts for 2% [3]. Consanguineous marriages increase the risk of AR dystrophies, which is prevalent in South Asian countries where the practice is most common, as well as in the Pakistani population [14,15]. The present study showed a higher consanguinity rate of 30% in the human study group.

The reported rates of nystagmus in humans range from 78.6% to 100%, with severe visual impairment being a predominant characteristic of LCA [16,17]. The results of this study found a 50% prevalence of nystagmus in humans, lower than previously published data. However, a higher percentage of severely impaired BCVA (70%) was found in cases of no LP, LP, and HM compared to previously published studies (57%) in patients with LCA-associated variants in the *GUCY2D* gene [18]. Comparing the visual acuity of the human group with the dog group, 100% of dogs had severe low visual acuity, failing both the cotton ball test and the avoidance of obstacles in photopic and scotopic conditions. This finding supports similar findings for the phenotypic expression of severe visual impairment due to a recessive variant in the *GUCY2D* gene [6].

Some patients in the human group showed RPE alteration, a finding that has been previously related to variant of this gene [19]. Color fundus photography exams showed subtle alterations, predominantly associated with arteriolar attenuation. As already well documented in the literature, the dissociation between function and morphology is particularly striking in human patients with LCA and was confirmed in the present study [20].

The present study also found subtle changes in the fundus photographs from the dog group that were better visualized on OCT as subtle outer retina changes, similar to the human group. The fundus images of the dog group abnormalities included slight changes in arteriolar attenuation, pigmentary mobilization, choroidosis, and possible variations in the tapetal reflectivity. The presence of NRD and hyperreflectivity of the tapetum, characteristic of the canine species and common in cases of progressive retinal atrophy, is a major difference due to the absence of tapeta in humans.

In humans, the ERGs were mostly undetectable but may maintain a residual rod response in some patients with *GUCY2D*-associated LCA [20,21]. ERG scans of humans showed significant a- and b-wave reduction, and in the dog group, virtually no response to the light stimulation of rods under scotopic and residual cone response in photopic conditions was observed. ERGs were extremely compromised in both groups, compatible with the recessive variant phenotype of the *GUCY2D* gene.

Human OCT scans show the compromised integrity of the ONL and ellipsoid zone in patients with LCA [16,20]. Although other studies reported mild outer retinal involvement with progression over the years [18,19,20,21], the human group included OCT scans of the macula from five patients that showed greater outer retinal involvement than in the previously published data. However, in humans, the OCT scans ranged from apparent normality, outer retinal irregularities, and focal ellipsoid zone atrophies to complete outer retinal atrophy. In the dog group, similar outer retinal changes were also found, including ellipsoid zone rarefaction, areas of outer retinal atrophy, and complete outer retinal atrophy in one dog (dog 10). Another study of APR in dogs associated with the *RPE65* variant showed, via OCT, a reduction in total retinal thickness in the affected group, with the main reduction in the outer retina and an increase in the thickness of the RPE [22].

In normal dog eyes subjected to OCT, the total retinal thickness of the superior sector (dorsal) was 204 μm and the inferior (ventral) sector was 178 μm. The total retinal thickness was significantly greater in the superior sector compared to the inferior (*p* = 0.010) [23]. Another study of the ONL in dogs showed that it rapidly decreases in thickness between 4 and 12 weeks of age, and the average thickness of the ONL of the superior retina is 58.9 ± 4.6 μm, compared to 52.4 ± 7.7 μm for the inferior retina [24]. Retinal OCTs in the dog group showed a reduction in ventral retinal thickness relative to dorsal retinal thickness in the entire group, with a reduction in the ONL thickness in only two dogs. In addition, OCT scans and fundus photography identified retinal *bullae* (similar to NRD in humans), which has also been observed in Whippet dogs affected with another form of inherited retinopathy [25]. Overall, the preservation of the ONL found in the dog group is similar to the OCT scans of human, in which the predominance of alterations is in the outer retina.

The involvement of the macula with foveal hypo-reflectivity is not suggestive of fluid NRD, but rather is a degenerative process featuring photoreceptor loss and the transient separation of the layers for subsequent focal retinal atrophy at that location. This finding is similar to the NRD in dogs—the bullae—and has been described previously in hereditary RD [26]. However, this was not identified in any of the human group patients of the present study.

In another study involving 31 dogs with a form of hereditary retinopathy, 58% of the affected dogs were male and the main fundoscopy findings were tapetal hyperreflectivity, diffuse vascular attenuation, hypopigmentation of the non-tapetal area, and pallor of the optic disc [27]. Three dogs affected by a different form of early onset hereditary retinopathy in the Shih Tzu breed showed similar results [28]. In the present study, the fundus photographs of the dogs showed very subtle changes similar to those already described in the literature. These data are also similar to those found on fundus photography of humans, with six patients presenting very close to normal (or only showing abnormalities with FAF).

The safety and preliminary efficacy of escalating doses of ATSN-101, a subretinal gene therapy for LCA type 1, were assessed in another study with significant improvements in retinal sensitivity observed in patients receiving the high dose [29]. Previous studies in animal models of LCA1 had demonstrated the restoration of rod and cone function via electroretinography following the administration of *GUCY2D* mediated by a recombinant adeno-associated virus serotype, underscoring the importance of animal models in research [29].

The limitation of the current study was the use of retrospective data. As inherent with all retrospective evaluations, the review of the data could be more robust if there was a greater continuity in the exams and better-quality images had been obtained.

Additional evidence from this study to support the rationale for the gene therapy model in dogs is the similarities demonstrated by ophthalmological and clinical examinations when comparing the two groups. This study demonstrates that the German Spitz dog animal model has clinical features similar to humans affected with LCA; these dogs exhibit subtle changes in fundus photographs, retinal thickness reduction with changes in the outer retina, important functional reduction determined by extreme visual impairment, and the reduction in a and b waves on ERG. Recently, a patent was published for gene therapy of the *GUCY2D* variant associated with LCA, consisting of a recombinant adenovirus vector containing nucleic acid for the production of guanylate cyclase. Restoration in a mouse model resulted in the improvement of the ERG by 45% for cones, and increased retinal organization on OCT compared to untreated mice [30].

This manuscript offers a unique perspective by presenting, within a single study, comprehensive data from an analogous form of disease in both humans and dogs, enabling a direct cross-species comparison. While previous studies have shown that dogs can serve as valuable models for Leber congenital amaurosis (LCA), our study strengthens this evidence by focusing on the same gene (GUCY2D) and the same inheritance pattern in both species.

A recent ARVO presentation by Beckwith-Cohen and colleagues demonstrates that gene augmentation therapy can achieve partial restoration of vision in dogs affected by *GUCY2D*-associated LCA [31]. Using an AAV-based vector to deliver a functional *GUCY2D* gene into the retinas of mature dogs, the researchers observed measurable improvements in visual function—evidence that even after retinal maturation, some photoreceptors remain rescuable. While the degree of functional recovery did not reach full normality, these results importantly validate the dog *GUCY2D* LCA model as a translational platform for preclinical gene therapy and underscore the potential for restoring vision in LCA patients [31]. We propose that gene therapy in the group of dogs with a spontaneous genetic variant would be an excellent model for the treatment of variants in the *GUCY2D* gene in both dogs and, potentially, humans.

## Figures and Tables

**Table 1:** *GUCY2D* autosomal recessive variants and clinical and ophthalmological findings. Details of the 10 patients affected by the AR variant in the *GUCY2D* gene and hereditary retinal dystrophy. Note the alleles affected. Patient 09 did not have complete data.

ID	Allele 1	Allele 2	Sex	Age (Years)	BCVA (Snellen Table)	History and Eye Exam	Macular Alteration
01	*c.1956+1G>A (paternal)*	*c.1245delT (maternal)*	M	10	No LP OU	3-month-old nystagmus (sibling of G08)	Foveal hyper-autofluorescence
08	*c.1956+1G>A (paternal)*	*c.1245delT (maternal)*	F	8	LP OU	Nystagmus, strabismus (sibling of G01)	Foveal hyper-autofluorescence
02	*c.2620G>A (p.Glu874Lys)*	*c.1A>G (p.Met1?)*	F	17	20/60 OU	Phenotype of cone dystrophy	Foveal hyper-autofluorescence
03	*c.1245delT (p.Phe415LeufsX73)*	*c.2598G>C (p.Lys866Asn)*	M	4	HM OU	Nystagmus and ET	No
04	*c.1052A>G (p.Tyr351Cys)*	*c.1052A>G (p.Tyr351Cys)*	F	55	20/80 OD–20/70 OS	Night blindness since childhood, myopia	Yes (hypertrophy of RPE foveal) OD, foveal hyper-autofluorescence OS
05	*c.1972C>T (p.His658Tyr)*	*c.1972C>T (p.His658Tyr)*	M	37	HM OU	Consanguineous parents Keratoconus, corneal leucoma in OS	Bull’s eye OU, ring hypo-autofluorescence around the fovea.
06	*c.1957-2A>G*	*c.1957-2A>G*	M	14	HM OD-LP OS	Consanguineous parents	No
07	*c.1343C>A (p.Ser448X)*	*c.1343C>A (p.Ser448X)*	M	10	No LP OU	Consanguineous parents Low VA, nystagmus from birth Hyperopia	No
09	*c.1343C>A (p.Ser448X)*	*c.1957-2A>G*	F	4	LP OU	Nystagmus, strabismus, low VA since 2 months	-
10	*c.2302C>T (p.Arg768Trp)*	*c.767A>C (p.Gln256Pro)*	F	23	20/150 OU	Change in macular brightness	Foveal RPE atrophy, foveal hyper-autofluorescence with hipo-autofluorescence areas OU

F: female, M: male, BCVA: best corrected visual acuity, OU: both eyes, LP: light perception, HM: hand movements, OD: right eye, OS: left eye, VA: visual acuity, ET: esotropia; RPE: retinal pigment epithelium.

**Table 2:** Details for the dog group. Details of the dogs affected by the same variant in the *GUCY2D* gene, sex, age at diagnosis, fundus photography, ERG, and OCT. Patient 12 did not have complete data.

ID	Gender	Age at Diagnosis (Months)	ERG Age (Months)	OCT Age (Months)
1.	M	3	3	
2.	M	3	3	32
3.	M	3	3	36
4.	F	5	5	
5.	F	22	9	
6.	M	9	9	
7.	M	3	3	15
8.	M	3	4	8
9.	F	3	4	8
10.	M	3	4	13
11.	M	4	4	
12.	M	15	15	
13.	F	2	2	4
14.	F	2	2	
15.	F	2	2	
16.	M	1.5	3	

F: female, M: male, m: months, OCT: optical coherence tomography, ERG: electroretinography.

**Table 3:** Clinical and ophthalmological comparison between human and dog cohorts with autosomal recessive *GUCY2D*-associated retinal dystrophy. Comparing the two groups, the majority are male and the presentation of the disease is early with extreme visual acuity impairment associated with absent cone and rod function documented by electroretinography. BCVA was considered extremely compromised in cases of no light perception (LP), LP, and no hand movements (HM) detection. Fundus photographs were analyzed and categorized by the same ophthalmologists (BLVG, FMF) with emphasis on the presence of arteriolar attenuation, venous attenuation, optic disc pallor, retinal macular changes, presence or absence of neurosensory retinal detachment (NRD), the area of the tapetum, and presence or absence of tapetal hyperreflectivity. AR: autosomal recessive, BCVA: best corrected visual acuity, ERG: electroretinography, RPE: retinal pigment epithelium, OCT: optical coherence tomography.

	Human Group	Dog Group
**Age**	4 to 55 years	2 to 22 months
**Autosomal inheritance**	100% AR	100% AR
**Extremely low BCVA**	70%	100%
**Nystagmus**	50%	81.3%
**ERG**	Absence of responses from the cones and rods	Absence of responses from the cones and rods
**OCT**	Reduced/absence reflectivity of the RPE and the ellipsoid zone	Reduced/absence reflectivity of the RPE and the ellipsoid zone; Presence NRD
**Fundus Photography**	Arteriolar attenuation in 20%; Increased tortuosity in 20%; Pink optic nerve and veins with normal caliber in 100%; RPE atrophy or hypertrophy or drusenoid deposits in 30%; Hyper-autofluorescence in 60%;	Slight arteriolar attenuation in 53.8%; Increased tortuosity in 23.1%; Pink optic nerve and veins with normal caliber in 100%; Slight pigmentary mobilization and some degree of choroidosis in 18.8%; Tapetum was hypoplasic in 61.53% and completely absent in 30.8%; Hyperreflexivity of the tapetum in 50%; NRD in 18.8%

## Data Availability

The datasets used and/or analyzed during the current study are available from the corresponding author on reasonable request due to privacy and ethical restrictions.

## Supplementary Materials

The following supporting information can be downloaded at https://www.mdpi.com/article/10.3390/vetsci12090879/s1; Figure S1: Ophthalmological findings of patients 04-06 in the human group; Figure S2: Ophthalmological findings of patients 07-08-10 in the human group; Figure S3: VF exams of the human group; Figure S4: Ophthalmological findings in the dog group.

## Abbreviations

The following abbreviations are used in this manuscript:

LCA	Leber’s congenital amaurosis
CRD	Cone-rod dystrophy
OCT	optical coherence tomography
ERG	electroretinogram
AR	autosomal recessive
AD	autosomal dominant
UFPR	Federal University of Paraná
BCVA	best corrected visual acuity
FAF	autofluorescence
VF	visual field
RD	retinal dystrophy
NRD	neurosensory retinal detachment
LP	light perception
HM	hand movements
RPE	retinal pigment epithelium
ONL	outer nuclear layer
