# Supplementary material for: GUCY2D-Associated Retinopathy: A Comparative Study Between Humans and German Spitz Dogs
Source: Vet Sci. 2025 Sep 11;12(9):879. doi: 10.3390/vetsci12090879 (PMC12474124; doi:10.3390/vetsci12090879)
Supplement: Supplementary file 1 [file vetsci-12-00879-s001.zip › vetsci-3712330-supplementary.pdf]

veterinary sciences

MDPI

Supplementary Material File

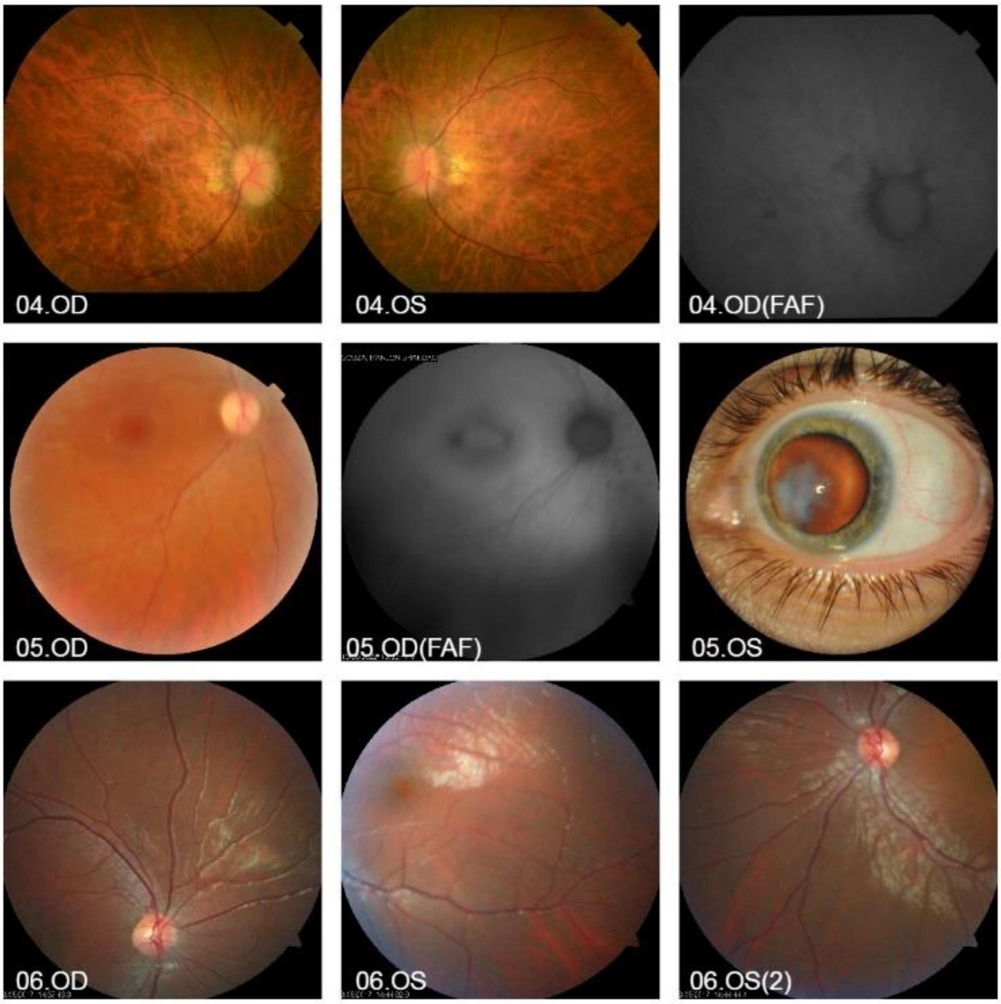

Supplementary Figure S1. Ophthalmological findings of patients 04-06 in the human group. 04. OD and 04.OS**.** On the color fundus photography foveal RPE hypertrophy/atrophy OD, choroidosis and arteriolar attenuation OU. 04.OD (FAF**).** In the auto fluorescence focal hypo-autofluorescence OD. 05.OD**.** On the color fundus photography, a maculopathy pattern in Bull's eye OD and arteriolar attenuation. 05. OD(FAF). In the auto fluorescence hyper-autofluorescence surrounded by hypo-autofluorescence surrounded by retinal mottling OD**.** 05.OS. In the photo of the anterior segment showing keratoconus and corneal leukoma in OS. 06.OD, OS and OS (2). On the color fundus photography without alterations. RPE retinal pigment epithelium. OU both eyes, OD right eye, OS left eye.

 https://doi.org/10.3390/vetsci12090879

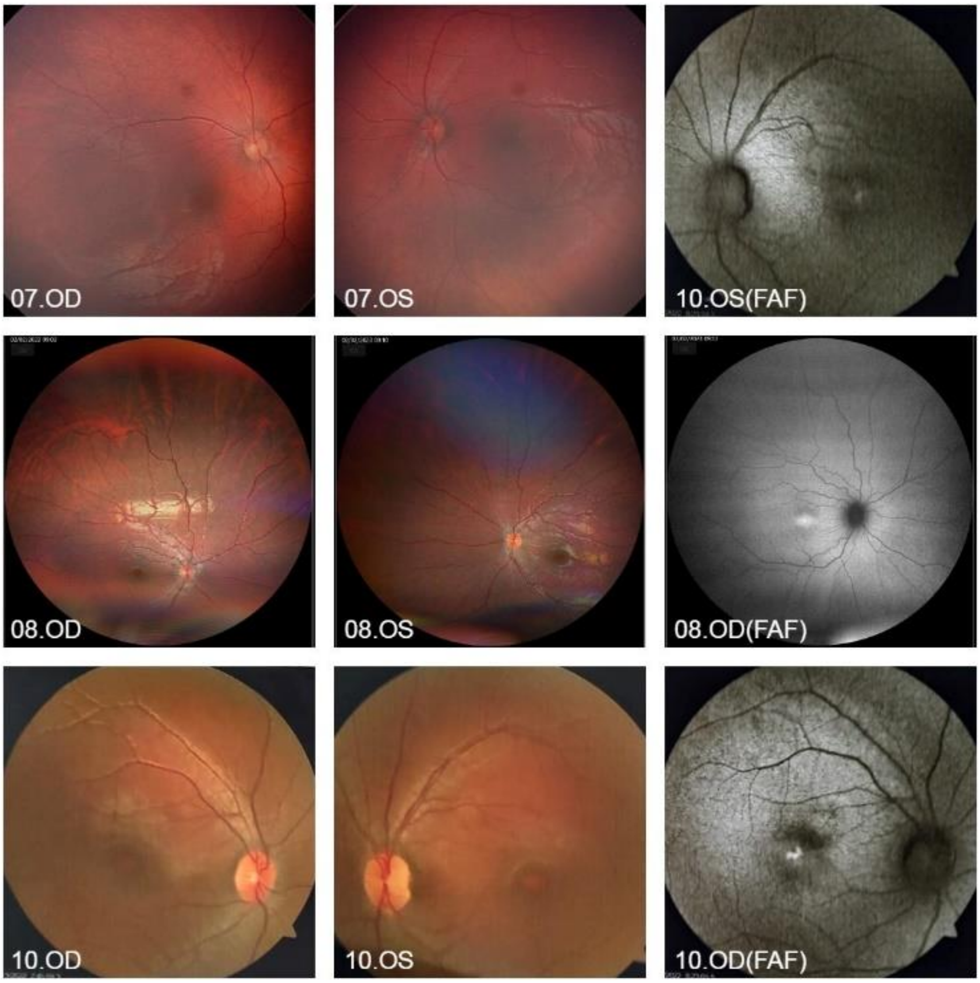

Supplementary Figure S2. Ophthalmological findings of patients 07-08-10 in the human group**. 07**. OD and 07.OS. On the color fundus photography without alterations OU. 10. OD(FAF) and OS(FAF). In the auto fluorescence foveal hyper-autofluorescence with some hipo-autofluorescence areas OU. 08.OD and OS. On the color fundus photography increased arteriolar tortuosity are present OU. 08. OD(FAF). In the auto fluorescence hyper-autofluorescence. 10.OD and OS. On the color fundus photography foveal RPE atrophy OU. RPE retinal pigment epithelium, OD right eye, OS left eye.

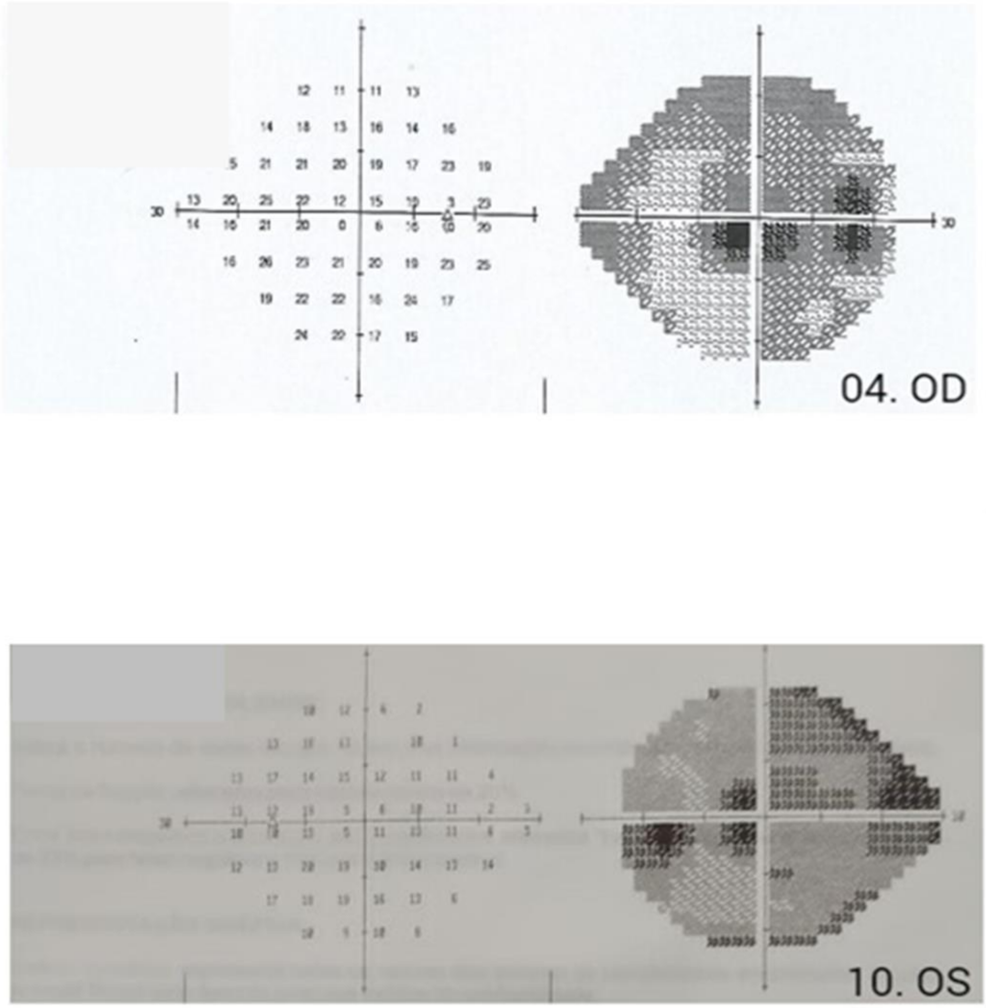

Supplementary Figure S3. VF exams of the human group. VF exams of patient 04 and 10 exhibits a significant reduction in sensitivity macula and decreased sensitivity in all quadrants. VF visual field.

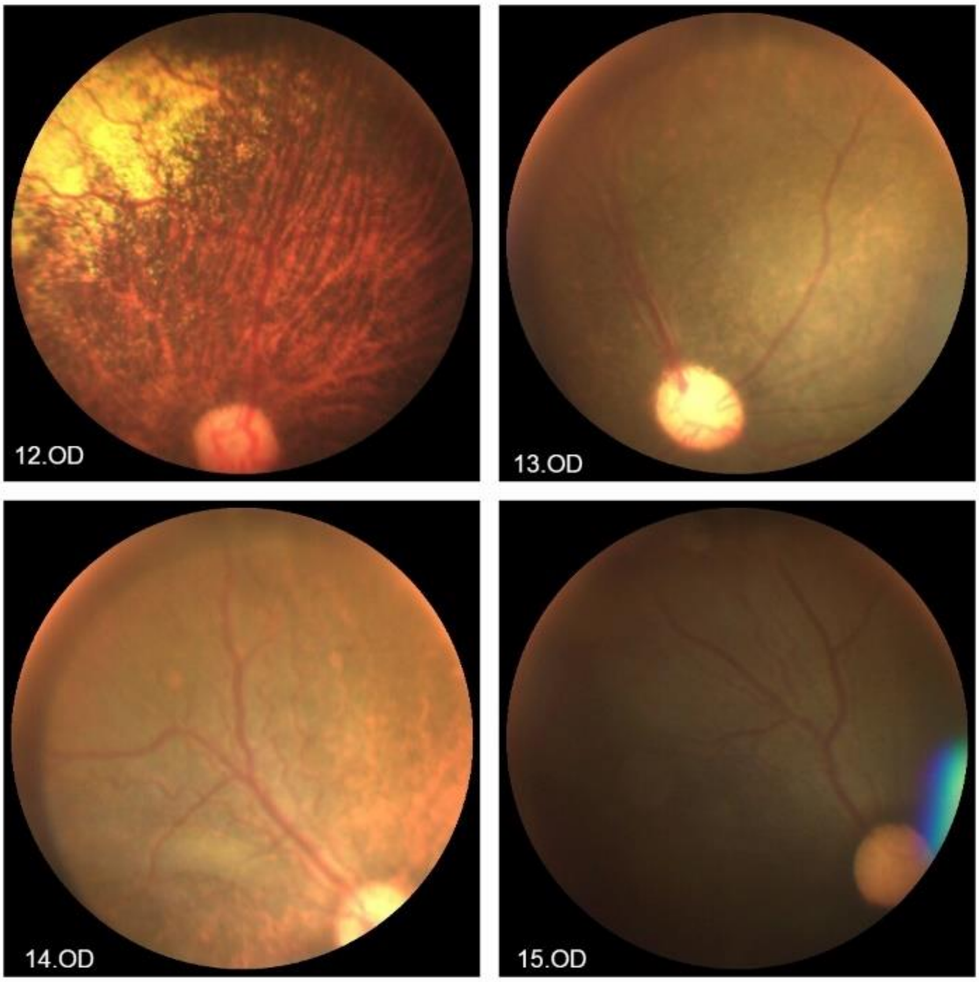

Supplementary Figure S4. Ophthalmological findings in the dog group. 12. OD**.** Arteriolar attenuation and increased tortuosity, tapetum hypoplasic and hyperreflexivity associated choroidosis were present in OD. 13.OD. Arteriolar attenuation, tapetum absent, slight pigmentary mobilization and choroidosis were present in OD. 14.OD. Tapetum absent, slight pigmentary mobilization and choroidosis were present in OD. 15.OD. Tapetum absent in OD**.** OS left eye, OD, right eye.
